# Direct Acting Antivirals in Patients with Chronic Hepatitis C and Down Syndrome

**DOI:** 10.1155/2016/2605302

**Published:** 2016-10-26

**Authors:** Eric R. Yoo, Ryan B. Perumpail, George Cholankeril, Aijaz Ahmed

**Affiliations:** ^1^Department of Medicine, University of Illinois College of Medicine, Chicago, IL, USA; ^2^Division of Gastroenterology and Hepatology, Stanford University School of Medicine, Stanford, CA, USA; ^3^Division of Gastroenterology and Hepatology, University of Tennessee Health Sciences Center, Memphis, TN, USA

## Abstract

Patients with Down syndrome who received blood transfusions, likely in conjunction with cardiothoracic surgery for congenital heart disease and prior to the implementation of blood-donor screening for hepatitis C virus infection, face a substantial risk of acquiring the infection. In the past, interferon-based therapy for chronic hepatitis C infection in patients with Down syndrome was noted to have lower efficacy and potentially higher risk of adverse effects. Recently, the treatment for chronic hepatitis C has been revolutionized with the introduction of interferon-free direct acting antivirals with favorable safety, tolerability, and efficacy profile. Based on our experiences, the newly approved sofosbuvir-based direct acting antiviral therapy is well tolerated and highly efficacious in this subpopulation of hepatitis C virus infected patients with Down syndrome.

## 1. Introduction

Prior to the implementation of blood-donor screening for hepatitis C virus infection, children who underwent cardiac surgery faced a substantial risk of acquiring the infection [1]. Chronic hepatitis C in patients with Down syndrome is usually a consequence of such childhood transfusions instituted in conjunction with cardiothoracic surgery for congenital heart disease [1–3]. To complicate matters further, an array of immunological disorders such as abnormal composition of peripheral blood lymphoid subsets, cellular dysfunction, and autoimmunity have been commonly noted in the setting of Down syndrome [4]. However, the majority of these patients do not manifest clear clinical features of immunological disorders. Despite these observations, limited experiences with interferon-based therapy in the setting of Down syndrome showed dismal results with none of the patients responding to treatment [5], raising the question whether immunological disturbances in patients with Down syndrome may result in poor performance of immune-dependent interferon therapy [4].

More recently, the treatment for chronic hepatitis C has been revolutionized with the introduction of interferon-free direct acting antivirals (DAA) with favorable safety, tolerability, and efficacy profile. Patients with chronic hepatitis C and Down syndrome form a unique group in which sofosbuvir- (SOF-) based DAA regimens may be promising and need further evaluation. We present our experience with the use of SOF-based regimens in this subpopulation of HCV-infected patients. Please note that the three patients presented below were monoinfected with chronic hepatitis C and with serologies negative for coinfection with hepatitis B virus or human immunodeficiency virus.

## 2. Case Presentations

### 2.1. Patient 1

Our first patient is a 27-year-old Caucasian man with trisomy 21, Down syndrome, and chronic hepatitis C virus, genotype 1b. He developed transfusion-related chronic hepatitis C, most likely following cardiothoracic surgery early in life for an endocardial cushion defect. Pretreatment laboratory data showed platelet count 98 × 10^3^/microL, prothrombin time 14.2 seconds, INR 1.2, aspartate aminotransferase 74 units per liter (U/L), alanine aminotransferase 103 U/L, alkaline phosphatase 104 U/L, normal gamma-glutamyl transferase, total bilirubin 0.7 milligrams per deciliter (mg/dL), and hepatitis C virus RNA level 1.23 million International Units (IU)/mL. His liver biopsy in 2009 showed grade 3 inflammation and stage III fibrosis. A computerized tomography scan of the abdomen showed a nodular cirrhotic liver with mild splenomegaly. No hypervascular discrete hepatic lesions were noted to suggest hepatocellular carcinoma. He failed to respond to combination antiviral therapy using pegylated interferon plus ribavirin. He was retreated with telaprevir, pegylated interferon, and ribavirin. He did not respond to second course of antiviral therapy. He developed severe adverse effects during the two courses of interferon-based antiviral therapy requiring the use of growth factors and transfusions. He underwent retreatment with interferon and ribavirin free oral DAA regimen using SOF and simeprevir for 12 weeks in 2014 and developed a sustained virological response at 6 months following completion of antiviral therapy. He remained aviremic and without any liver related symptoms. His liver enzymes normalized during antiviral therapy and remained thrombocytopenic. He has been recommended to undergo surveillance for hepatocellular carcinoma every 6 months.

### 2.2. Patient 2

Our second patient is a 29-year-old Caucasian woman with Down syndrome. She underwent open heart surgery at age 6 months for an endocardial cushion defect in 1986. She required blood transfusions resulting in chronic hepatitis C virus RNA level 0.9 million IU/mL, genotype 2a. She has never undergone a liver biopsy. She did not demonstrate any stigmata of portal hypertension or advance liver disease. She underwent her first course of antiviral therapy for 12 weeks using SOF and ribavirin 400 mg twice a day in 2015. She tolerated the treatment well and developed minimal adverse effects during antiviral therapy. According to her caregiver, she was minimally fatigued during antiviral therapy; however, there were no major adverse effects. Her aminotransferases, alkaline phosphatase, gamma-glutamyl transferase, total bilirubin, platelets, and coagulation studies remained normal prior to and following completion of antiviral therapy. She was able to continue her daily chores and attend school. Our patient was noted to have undetectable levels of hepatitis C virus on completion of the antiviral, and 6 months later she developed a sustained virological response.

### 2.3. Patient 3

Our third patient is a 53-year-old Caucasian man with chronic hepatitis C, genotype 1b. He has been noted to have persistent thrombocytopenia consistent with portal hypertension and suggestive of advanced stage III to IV hepatic fibrosis. Pretreatment laboratory data showed platelet count 97 × 10^3^/microL, coagulation studies normal, aspartate aminotransferase 54 U/L, alanine aminotransferase 49 U/L, alkaline phosphatase 189 U/L, gamma-glutamyl transferase normal, total bilirubin 0.5 mg/dL, and hepatitis C virus RNA level 27.8 million IU/mL. He did not undergo a liver biopsy. He underwent cardiothoracic surgery as a child and received transfusions leading to chronic hepatitis C. He was treated with SOF and ledipasvir combination therapy for 12 weeks in 2015. He developed a sustained virological response with undetectable levels of hepatitis C virus at 24 weeks following completion of antiviral therapy. The abnormalities in liver enzymes resolved within the first 8 weeks of antiviral therapy. He was asked to continue surveillance for hepatocellular carcinoma on a 6-monthly basis due to underlying advance liver disease (stage III to IV fibrosis).

## 3. Discussion

In our case series we report successful response rates to SOF-based DAA therapy in patients with Down syndrome. A detailed discussion was conducted with the court-appointed legal guardian—parents, family, and support staff members of the patients. In all three cases, treatment inquiry and request were initiated by the legal guardian of the patient. Efficacy data, side effect profile, treatment duration, black box warnings, and absolute contraindications noted in the package insert for SOF-based treatments were reviewed. Limitations of off-label therapy were also reviewed. Patients were very closely monitored during the therapy by the primary care provider with immediate access to the medical team. The three patients tolerated the therapy without any minor or major adverse effects and they did not experience any alterations in their quality of life, conducting their daily chores without interruption. They complained of antiviral therapy due to an already established firm support system provided by the primary care provider at home or nursing facility.

We did not experience any clinical evidence of immunological dysfunction in the three patients [4]. No new symptoms were reported during or after the SOF-based DAA therapy suspicious of immune-mediated disorder including skin rash, arthralgia, visual disturbances, infections, diarrhea, urinary complaints, and so forth. No new behavioral changes or psychiatric symptoms were reported. Two out of three patients demonstrated evidence of advance liver disease and were placed in a 6-monthly surveillance program for hepatocellular carcinoma. It is prudent to perform a comprehensive evaluation for advance fibrosis with patients with chronic hepatitis C and Down syndrome during their initial evaluation, as most of these patients were infected with hepatitis C virus prior to 1990 when screening protocols were not instituted by most blood banks in the United States. It may be challenging to perform liver biopsy in patients with Down syndrome due to pain associated with the procedure and the high level of cooperation needed from the patient. However, the recent availability of several noninvasive tests used to assess hepatic fibrosis may overcome these issues and be invaluable in this patient population.

## 4. Conclusion

Previously, interferon-based therapy for chronic hepatitis C infection in patients with Down syndrome was noted to have lower efficacy and potentially higher risk of adverse effects. However, based on our experience, the newly approved SOF-based antiviral therapy is well tolerated and highly efficacious in this patient population. We recommend that these patients with Down syndrome and chronic hepatitis C infection undergo close monitoring during their DAA therapy.
